# Dynamics of Collective Decision Making of Honeybees in Complex Temperature Fields

**DOI:** 10.1371/journal.pone.0076250

**Published:** 2013-10-16

**Authors:** Martina Szopek, Thomas Schmickl, Ronald Thenius, Gerald Radspieler, Karl Crailsheim

**Affiliations:** Artificial Life Laboratory of the Department of Zoology, Karl-Franzens University Graz, Graz, Austria; University of Sheffield, United Kingdom

## Abstract

Endothermic heat production is a crucial evolutionary adaptation that is, amongst others, responsible for the great success of honeybees. Endothermy ensures the survival of the colonies in harsh environments and is involved in the maintenance of the brood nest temperature, which is fundamental for the breeding and further development of healthy individuals and thus the foraging and reproduction success of this species. Freshly emerged honeybees are not yet able to produce heat endothermically and thus developed behavioural patterns that result in the location of these young bees within the warm brood nest where they further develop and perform tasks for the colony. Previous studies showed that groups of young ectothermic honeybees exposed to a temperature gradient collectively aggregate at the optimal place with their preferred temperature of 36°C but most single bees do not locate themselves at the optimum. In this work we further investigate the behavioural patterns that lead to this collective thermotaxis. We tested single and groups of young bees concerning their ability to discriminate a local from a global temperature optimum and, for groups of bees, analysed the speed of the decision making process as well as density dependent effects by varying group sizes. We found that the majority of tested single bees do not locate themselves at the optimum whereas sufficiently large groups of bees are able to collectively discriminate a suboptimal temperature spot and aggregate at 36°C. Larger groups decide faster than smaller ones, but in larger groups a higher percentage of bees may switch to the sub-optimum due to crowding effects. We show that the collective thermotaxis is a simple but well evolved, scalable and robust social behaviour that enables the collective of bees to perform complex tasks despite the limited abilities of each individual.

## Introduction

Temperature is one of the most important environmental factors for insects, as most insect species can only survive and reproduce within narrow temperature ranges [1], [2]. Whereas most insects can regulate their body temperature only indirectly by the environmental temperature they locate themselves at, the Western honeybee (*Apis mellifera*) is able to produce heat endothermically [3]–[5]. This adaptation is one main reason for the wide distribution of this species as it allows the whole colony to survive cold winters [6], [7]. The endothermic heat production and regulation of hive temperatures enables honeybee colonies to start with brood-rearing in mid-winter. As the period where the maximum amount of food is available is short, this adaptation increases the work force and maximises the food intake and in turn ensures the survival of the colony in the following winter [8].

Active heating is one crucial behavioural adaptation that honeybees use to keep the temperature of the brood nest within a narrow temperature range from 32°C to 36°C [9], [10]. Such collective control of brood nest temperature is of great importance for an optimal larval and pupal development [11]–[13]. Most individuals of a colony, both worker bees and drones, contribute to this social thermogenesis, whereas freshly emerged bees are not yet able to do so [4], [14]. Such young bees are located within the warmer areas of the hive [15], the brood nest. There they find the appropriate thermal conditions and pheromone supply for their further development.

The ability to actively produce heat is hardly developed up to an age of 36 hours and is fully developed at an age of 5 days [16]. As these young ectothermic bees have not yet developed the physiological capabilities of endothermic heat production, they therefore have no alternatives to pursue the previously mentioned phylogenetically older strategy of positioning themselves in areas with the appropriate environmental conditions. To determine the temperature preferendum of those young ectothermic bees, Heran [17] tested such young bees in a temperature gradient apparatus, basically a more-or-less one-dimensional metal rail heated on one side and cooled on the other providing a rather steep and linear thermal gradient ranging from approx. 10°C to 50°C [18], [19]. In this device the young bees tend to locate themselves at their preferred temperature of 36°C, according to the temperature in the brood nest.

Surprisingly, comparable experiments from Kernbach et al. [20] conducted in a broader two-dimensional temperature arena with a thermal gradient more similar to the thermal conditions in the brood nest showed that groups of bees are able to find the preferred temperature, whereas the majority of single bees is not. They stated that for the collective thermotaxis the decisive cue must be the event of two bees stopping after meeting each other in the arena while performing a more or less randomized walk. They also found a positive correlation between resting time after the stop and the local temperature. The self-positioning of the young bees in the brood nest does not only support the development of the young bees. Seeley [21] found, that the tasks performed by different castes of the bee colony map on particular nest regions and that there is an efficiency compromise between the location and the performance of a task. Freshly emerged bees perform a single task in the brood nest, which is the cleaning of the cells. The collective thermotactic behaviour leads to the aggregation of these bees at their working place without the need of an additional coordination effort or the need of establishing an extra communication channel. This way the colony maximises the task performance very energy efficiently.

Thus, the observed collective thermotaxis in honeybees can be considered as a social behaviour that maximises energy efficiency by supporting collective decision making. Such collective behaviours that emerge from a group of social animals [22] implies collective decision making processes like nest site or food source selection as well as complex tasks like division of labour, nest construction, colonial thermoregulation or formation of transportation networks [23]–[28]. To investigate collective decision making processes, experiments in which groups of animals have to choose between two or multiple options were conducted with various species. Halloy et al. [29] examined the collective shelter selection of cockroaches, also studied by [30]–[32]. Interestingly, also in this species the collective aggregation under shelters is based on the modulation of resting time, similar to our bees’ behaviour. Other examples are comparable collective behaviours observed in the ant *Messor barbarus* during aggregation site selection [33], exploitation of food sources by the Pharaoh’s ant [34], aggregation behaviour of *Blattella germanica* cockroaches [30], path selection in the ant *Lasius niger* [35], nectar source selction of foraging honeybees (*Apis mellifera*) [24] or the nest site selection of swarming honeybees (*Apis mellifera*) [36].

The aim of this paper is to further investigate the social aspects of collective thermotaxis in honeybees based on the findings of Kernbach et al. [20]. For our experiments with single bees and groups of bees we went beyond a rather simple gradient with one optimum and laid our focus on the collective decision making of groups of bees in a more complex thermal environment. We define a collective decision making as a decision made by the group of bees in order to locate the majority of the swarm members in the global optimal temperature zone. We see this behaviour as being a collective feature if single bees in the same temperature field will be less often able to choose the optimum compared to the fraction of the swarm that is able to do so when the bees run in parallel. Experiments with single bees were conducted to compare their thermotactic abilities to the collective thermotaxis of the groups. We developed a new arena set-up where we are able to generate a thermal gradient with two optima of different attractiveness for bees. The emanating questions are manifold: How will the bees be distributed in the gradient? The bees could for example split up equally between the two different optima or show a preference for one optimum. An equal distribution of the bees between the two optima, although they are of different attractiveness, would give evidence that the bees perform an uphill walk. We assume that the bees perform collective thermotaxis and thus show a strong preference for the optimal spot, as they should wait longer in the optimum than in the sub-optimum. Is there a correlation between groups size and distribution of the bees between the optima? Different densities may lead to different behavioural patterns, for example there could be a minimum number of bees that is needed to perform the collective thermotaxis. Is the speed of the decision making process group size related? As the meeting probability is increased with increasing group size, we propose that larger groups decide faster than smaller ones. Additionally we examined the aggregation behaviour in homogeneous environments. We were interested in whether the bees do choose their locations independently. An alternative hypothesis would be that the bees choose their location based on the local temperature. However, this factor was excluded by testing the bee behaviour in an uniform temperature field. Another alternative hypothesis is that the bees choose their location based on the local number or density of other bees. Additionally, we examined whether the bees form one big aggregation also in absence of a complex thermal environment and thus the aggregation in an optimal temperature spot could happen by chance frequently. In this work we give answer to these questions and show that the collective decision making of young honeybees is a collective capability of the honeybee society arising from simple mechanisms of social interaction.

## Materials and Methods

### Animals

We conducted the experiments presented here with honeybees (*Apis mellifera*) aged from 1 h to 30 h, which are still ectothermic at this age [16]. The bees were removed from the colony as sealed pupae and hatched in incubators at 35°C and at a relative humidity of 60%. Freshly emerged bees were brushed from the combs and housed in ventilated plastic boxes and fed honey *ad libitum* until the start of the experiment on the same day. Bees with any obvious external damage (e.g. missing or mutilated antennae, legs or wings) were discarded. Each individual was tested only once and after the experimental runs all bees were returned to their original hives.

### Temperature Arena

All experiments were carried out at the Artificial Life Laboratory of the Department of Zoology at the Karl-Franzens-University in Graz, Austria.

**Figure 1:**
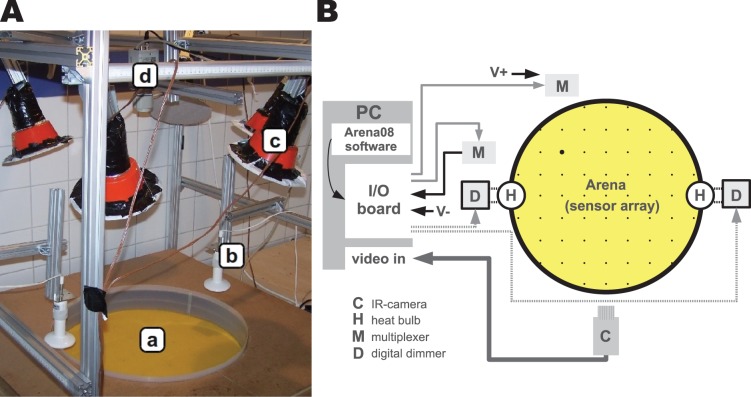
Experimental set-up. A) Overview over the experimental set-up, consisting of a circular wax arena (a) and two heat-bulbs (b) which generate the thermal gradient. A set of IR-emitters (c) generate light (not visible for honeybees) to allow observations with an IR-Sensible camera (d). B) Schematic drawing of the experimental set-up: Main control unit of the experimental set-up is an standard PC, including a state-of-the-art I/O board. This I/O board controls two multiplexers M, that control the sensor-array in the arena-floor. The data from the sensors is fed back into the I/O-board. Further the IO board controls the two heat-bulbs H (using two digital dimmers D). The image recoded by the camera C is fed into the PC and stored on mass storage devices. Gray solid lines indicate control lines for the multiplexers, black solid lines indicate the data-line from the temperature sensors, grey dashed lines indicate control-lines for the digital dimmers. The bold solid line indicates the video-line from the camera to the PC.

We developed a temperature arena with a diameter of 60 cm (Fig. 1a). A plastic wall coated with Teflon spray (Kroon Oil Tefspray, Almelo, The Netherlands) prevents the bees from climbing out. The arena floor consists of a perspex plate with 61 recessed temperature sensor modules (sensor: NTC SEMI833-ET, JBL GmbH & Co. KG, Löffingen, Germany; diode: 1N4148, Diotec Semiconductor AG, Heitersheim, Germany) covered with bees wax sheets. We exchanged these wax sheets after each trial to remove possible pheromone marks. Three additional temperature sensor modules measured the ambient temperature of the room. To compensate fluctuating ambient temperatures that would influence the desired gradient we regulated the air temperature in the experimental room. We heated the room with a radiator (EOS 7420z 2000W) connected to a thermostat or cooled it with a portable air conditioner (Sanyo SA-P61G5). This devices were also used to generate a stable ambient temperature when experiments with a homogeneous temperature distribution in the arena were conducted. We deactivated these devices during the experiments in order to avoid any irritation of the bees due to air-currents and noise/vibration. To generate the thermal gradient we used two ceramic heating lamps (ReptilHeat 60 W, JBL GmbH & Co. KG, Neuhofen, Germany) mounted above the arena. A standard computer controlled the two digital dimmers (Velleman K8064, Gavere, Belgium) of the heating lamps by using the data of the temperature sensors (Fig. 1b). This sensor-actuator system combined with adequate control software allowed us to generate a stable thermal gradient. To exclude visual cues and to stay closer to hive conditions we conducted all experiments under infra-red light which is not visible to bees [37], [38]. Therefore, we mounted IR filters (Schott & Gen. IR Filter 22 cm, Mainz, Germany) in front of halogen lamps. We filmed the trials with an IR-sensitive camera (WV-BP330/GE, Panasonic, Osaka, Japan) mounted above the arena and recorded it on an ME 1000 sMM Multimedia Center (Gerhard Witter GmbH, Schönberg, Germany (vendor)). We used dedicated software to read and log the temperature data and to control the gradient.

### Experiments

**Figure 2:**
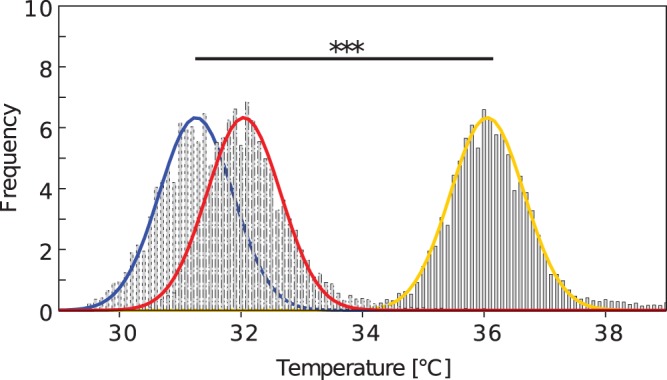
Temperature distribution of the different target temperatures in the arena in experiments with a complex gradient. Measurements of the central sensor of each zone are averaged over all experiments. The temperature distributions are significantly different between all three zones (p<0.05, T test). The targeted temperatures were as follows: outside the zones (blue): 31±1°C; right zone (red): 32±1°C; left zone (yellow): 36±1°C.

We carried out three experimental series. In the first experiment we tested single bees in a complex thermal environment with one optimal (optimum, 36°C) and one sub-optimal (sub-optimum, 32°C) spot. The average temperature distribution for the complex thermal environment in the different zones is shown in figure 2. In the second experiment differently sized groups of young bees were tested in the same complex thermal environment. In the third experiment differently sized groups were tested in homogeneous temperature distributions. In all trials the bees were introduced in the centre of the arena and every run lasted for 30 minutes.

#### Complex gradient with optimum and sub-optimum

**Figure 3:**
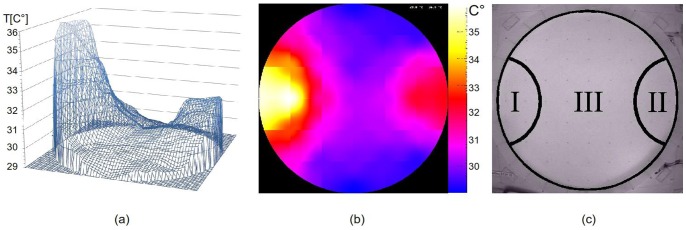
Complex thermal gradient and evaluation zones. (A) 3-dimensional and (B) colour-coded representation of the thermal gradient used for the discrimination experiments. The targeted temperatures were as follows: optimum (left zone) 36°C, centre of the arena (outside the zones) 31°C and sub-optimum (right zone) 32°C. (C) Picture from the arena with marked zones used for evaluation: I) optimum: left zone (11,2% of the total area), II) sub-optimum: right zone (11,2% of the total area), III) outside the zones (77,6% of the total area).

We introduced the bees in the arena with a complex thermal gradient where they had to choose between an optimum and a sub-optimum. We tested single bees (N = 10) and groups of 6, 24, 64 and 128 bees (N = 8/group size) in a gradient containing one optimal spot of 36±1°C and one sub-optimal spot of 32±1°C. The temperature in the centre of the arena was at 31±1°C (Fig. 3a,b).

#### Homogeneous temperature distribution

By introducing groups of bees in the arena with a homogeneous temperature distribution we examined the zonation of the bees in absence of a thermal gradient and compared it to the uniform distribution model (UDM, see “Materials and Methods” in the subsection “Data analysis”). Furthermore we determined the cluster distribution. We conducted experiments with groups of 24 and 128 bees. We examined the bees’ behaviour at 29±1°C (N = 10/group size) and at 36±1°C (N = 9 for 24 bees; N = 8 for 128 bees).

### Data Analysis

#### Evaluation zones

To evaluate the distribution of the bees in the arena we defined two equally sized zones beneath the heat lamps, corresponding to the area covered by the optimal and the sub-optimal temperature spot (each 11.2% of the total area of the arena, Fig. 3c). The left zone represents the area of optimal temperature, the right zone represents the area of sub-optimal temperature. We determined the number of bees in the left zone, the right zone and outside the zones at minute 30 on a still image of the recording of every trial. A bee was assigned to a zone when its thorax was mostly within the zone. A total of 79 trials were evaluated in this study.

#### Uniform distribution model – UDM

The “uniform distribution model” hypothesises that the bees ignore the local temperature and other bees. The predicted percentage of bees in each of the three evaluation zones is therefore equivalent to the size of these zones. A deviation of the observed values from the values predicted by the UDM indicates that the bees do not choose their locations independently.

The predicted values of the uniform distribution model correlates with the area covered by the different zones (11.2% for the optimal and the sub-optimal temperature spot, respectively, and 77.6% for the area outside the two zones).

#### Attraction Fields Model – AFM

The “attraction fields model” is used to predict the expected distribution of the bees in the three zones based on the hypothesis that all bees choose their location individually in the temperature field by just performing an uphill gradient walk. It also holds for the hypothesis that bees individually perform a random walk and just rest in warm areas as an individual decision and without any interference with other local bees. Also a combination of both hypotheses, that is some bees locate at the gradient maxima at an uphill walk and the other bees would either follow them or meet them after a random walk, leads to the same prediction made by the AFM.

We determined the data for the AFM by first identifying all sensor values in the arena above a certain threshold temperature (31.5°C). To determine the attraction fields for the optimum and the sub-optimum we assigned all sensor values above this threshold on the left side to the optimum and all sensor values on the right side of the arena to the sub-optimum. According to that the predicted percentage of bees is 59% in the optimum and 41% in the sub-optimum, with no bees expected outside the optima.

A deviation between the predictions of the AFM and the observed data indicates that the bees perform an alternative strategy which is not purely individual-decision making and also not collective aggregation around such pre-conquered places identified by a few uphill-walk capable leaders. Such an alternative strategy would require a modulation of the aggregation dynamics, that is either a modulation of the probability to join a cluster or a modulation of the time spent in such a cluster. Potential candidate factors for such a modulation are local temperature and/or local group density or group size.

#### Collective decision and speed of decison making

To determine whether a group of bees made a statistically significant decision for one of the two optima, that is the question whether or not the individual choice differed significantly from a random choice, we used a binomial test: The rationale is to test the measured distribution of bees against an individual random choice based on N independent and random (equal probability for optimal and suboptimal spot) decisions. The null hypothesis is therefore a binomial distribution of all possible combinations and we chose an occurrence probability of p<0.05 and indicating a non-random and thus statistically significant choice. For this analysis we identified all bees which are in either the optimum or the sub-optimum after 30 minutes, excluded the bees which stayed outside the optimum and sub-optimum in the middle of the arena, and determined the threshold number of bees which are sufficient in each run to classify this experimental result as a statistically significant decision. Due to the nature of the binomial function the percentage of bees needed for a statistically significant decision depends on the number of individuals involved in the decision making process. Further information about this method can be found in the supporting online material of [29].

To determine the time until the threshold for a significant decision was reached, further called “speed of decision making”, we analysed the recordings in one-minute intervals until a sufficient number of bees, defined by the method described above, was reached and stable for at least three consecutive intervals. We then further analysed the one minute interval when the threshold was first reached in one-second intervals to determine the point of time when the sufficient number of bees for a statistically significant decision was reached.

#### Waiting time

To verify the positive correlation between the local temperature and the waiting time of a bee after an encounter found by [20] for our set-up, we chose random bees of every trial with groups of bees in the complex thermal gradient and measured the time the bees stopped after encountering another bee or the arena wall in each of the three temperature zones.

#### Cluster analysis

For trials with homogeneous temperature distributions we determined the number and size of clusters at the end of each experiment in minute 30. We counted the number of: single bees, small groups (2–3 bees) and big groups (>3 bees).

## Results

### Waiting Time in Correlation with Local Temperature

**Figure 4:**
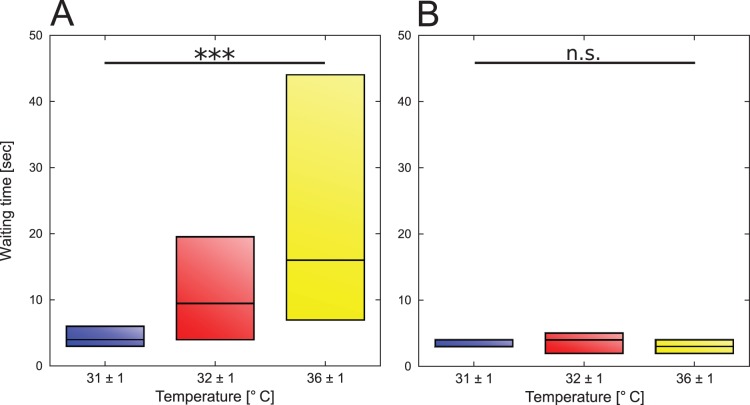
Waiting time after contact. Shown is the median waiting time (incl. quartiles) in the three different temperature zones after contact with (A) another bee (*r_s_* = 0.5025; p<0.01) and with (B) the arena wall (*r_s_* = 0.0448; p>0.05) (N of contacts = 720).

In total 720 contacts of bees with other bees or the arena wall were evaluated. The median waiting time after meeting another bee was 4 sec outside the zones, 9.5 sec in sub-optimum and 17.5 sec in the optimum. After encountering the wall the waiting time was 3 sec outside the zones, 4 sec in the sub-optimum and 3 sec in the optimum (Fig. 4a,b). The waiting time of a bee after meeting another bee was positively correlated with the local temperature (Fig. 4a, rs  =  0.5025; p<0.01) and was significantly different between the three zones. In contrast, the waiting time after encountering the arena wall was not correlated to the temperature and did not differ significantly between the three zones (Fig. 4b, rs  =  0.0448; p>0.05).

### Honeybee Decision making in Complex Gradients

**Figure 5:**
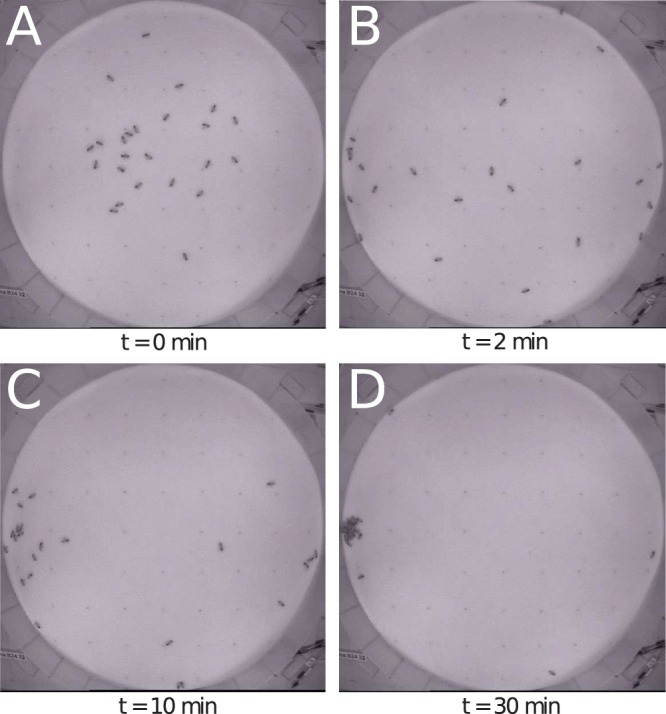
Pictures of an exemplary experimental run at different times. (A) minute 0: start distribution, (B) minute 2: dispersal, (C) minute 10: start of aggregation in optimum, (D) minute 30: aggregation at 36±1°C. The corresponding complex gradient is depicted in figure 3.

**Figure 6:**
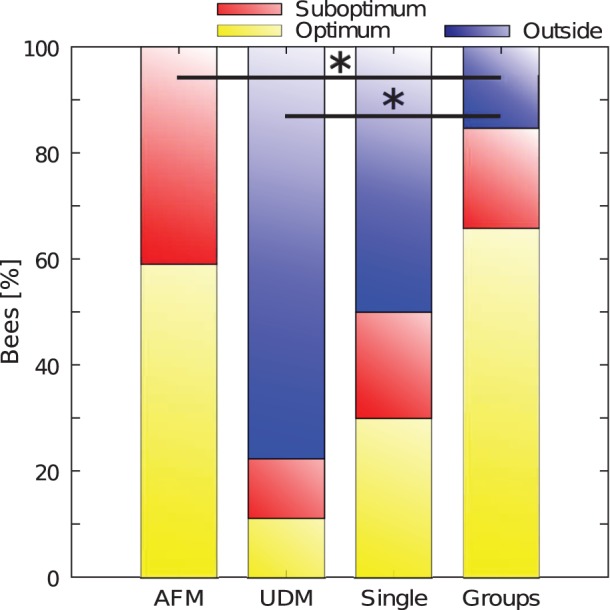
Comparison of the data from experiments to the attraction fields model (AFM) and the uniform distribution model (UDM). Shown is the percentage of single bees and groups of bees (all group sizes) in the optimum, sub-optimum and outside the optima compared to the AFM (threshold 31.5±1°C; 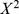-test p<0.05) and to the UDM (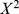-test p<0.05).

**Figure 7:**
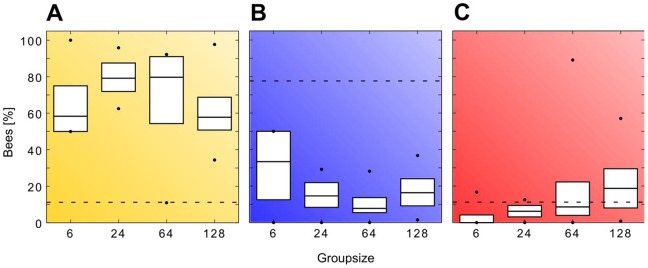
Results of the experiments with complex gradients. Shown is the percentage of bees (median, quartiles, minimum and maximum) a) in the left zone (36±1°C), b) outside the zones (31±1°C) and c) in the right zone (32±1°C) at the different group sizes (N = 8/group size). The dashed line represents the percentage of bees predicted by the UDM.

In these experiments single bees and differently sized groups of bees were introduced into the arena and had to choose between an optimal place of 36°C and a sub-optimal place of 32°C. For pictures of an exemplary experiment with a group of 24 bees please see figure 5. To answer the question whether or not the thermal gradient influences the spatial distribution of the bees, we analysed the zonation of the bees in the arena and compared it to the predicted zonation of the uniform distribution model (UDM) and the attraction fields model (AFM). Only 30% of the single bees located themselves at the optimum, 20% were in the sub-optimum and the ramaining 50% were located outside of the optima. The distribution of the bees including all trials did differ significantly from the UDM (p<0.05, 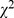 test, Fig. 6) and did not differ from the AFM (p<0.05, 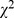 test, Fig. 6). We found a strong influence of the thermal gradient on the dispersal of groups of bees in the arena, as the distribution of the bees deviated significantly from the UDM when compared with every single trial as well as when compared to the pooled data including all trials of all group sizes (Fig. 6, p<0.05, 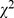 test). The bees clearly showed a preference for the optimal temperature spot, as the median percentage of bees there was significantly higher than the median percentage of bees located at the sub-optimal temperature spot within each group size (Fig. 7, p<0.05, Kruskal-Wallis test). No significant difference was found between the median percentage of bees at the optimal temperature spot in the different group sizes (Fig. 7a, p>0.05, Kruskal-Wallis test), whereas the median percentage of bees at the sub-optimal temperature spot increased with group size (Kendalls tau 0.3995; p = 0.0068). We found that at the sub-optimal temperature spot the median percentage in groups of 6 bees differed significantly from the median percentage in groups of 64 and 128 bees (Fig. 7c). Furthermore we compared the distribution of all bees from trials with groups of bees to the distribution of single bees and the predicted values for the AFM. Both, the distribution of groups of bees and the distribution of single bees, was significantly different from the AFM (p<0.05, 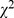 test, Fig. 6).

**Figure 8:**
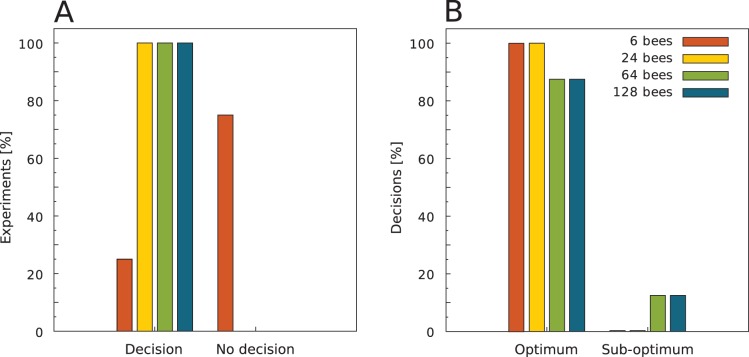
Collective decision in the four different group sizes. (A) The percentage of experiments with statistically significant collective decisions and (B) the percentage of the decisions for either the optimum or the sub-optimum is shown for all four group sizes. Note that (B) only includes data for when a collective decision was made by the bees.

With the first analysis we were able to determine the preference of the bees for the optimal temperature spot. However, with this analysis we could not determine a statistically significant measure whether or not the bees made a decision. Therefore we used a statistical model approach: We determined the number of bees at the optimal temperature spot for each run that are necessary to classify the observed behaviour as a statistically significant case of collective decision making. We have described the used statistical method in “Materials and Methods” in the subsection “Data analysis”. We found that groups of 24, 64 and 128 bees made a statistically significant choice in all runs whereas groups of 6 bees were able to decide collectively in one fourth of all experiments (N = 8 experimental runs/group size; Fig. 8a). Compared to the first analysis, where we took no account on a decision threshold it showed that in experiments with groups of 6 bees the distribution of the bees deviated from the predicted distribution for the UDM and the percentage of bees in the optimum exceeded the predicted value for the UDM (Fig. 7), but in most cases it may arise from random choices of the individual bees. If groups of 6 and 24 bees reached the threshold for a decision they collectively chose the optimum. In groups of 64 and 128 bees 87.5% of the decisions were made for the optimal spot, and 12.5% of the decisions were made for the sub-optimal spot (Fig. 8b).

#### Process over time and speed of honeybee decision making in complex gradients

**Figure 9:**
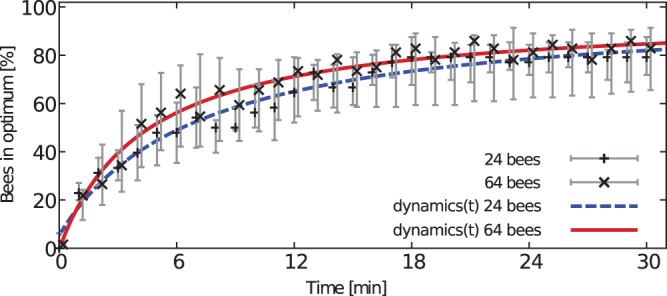
Aggregation process over time. The graph shows the decision making process over the whole experimental time of 30 minutes. Depicted is the percentage of bees (median and quartiles) in the optimum for groups of 24 and 64 bees. The curves were fitted according to the saturation function 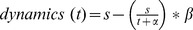.



The results concerning the zonation of the bees showed, that there is no difference in the different group sizes concerning the percentage of bees located in the optimum at the end of the experiment. Although the final outcome was the same in all group sizes, we assumed that there might be differences during the aggregation process due to the different densities. Therefore we analysed the aggregation process over time for the percentage of bees in the optimum for two different group sizes (24 and 64 bees, Fig. 9). We used a non-linear regression model, according to the equation.

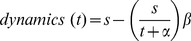
(1)whereby 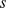 [percentage of bees] represents the saturation maximum for an infinite runtime and 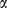 [minutes] and 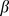 [minutes^−1^] represent free variables. For 24 bees we found that 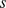 = 100%, 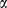 = 7.05 minutes and 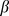 = 0.15 minutes^−1^. For 64 bees we found that 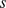 = 97%, 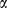 = 4.33 minutes and 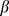 = 0.23 minutes^−1^. The non-linear regression model predicts, that nearly all bees (100% and 97% for groups of 24 bees and 64 bees respectively) gather at the optimum, in case of an infinite runtime.

**Figure 10:**
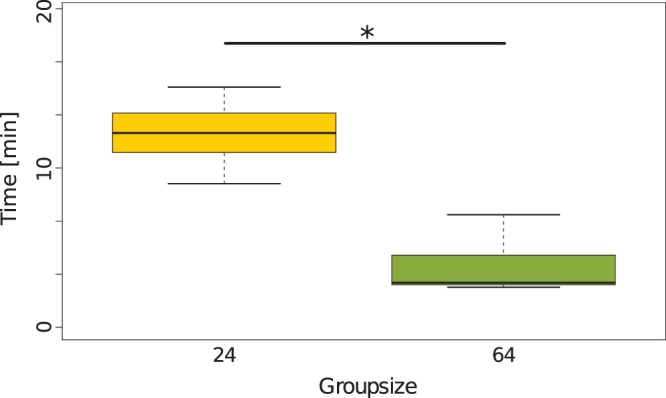
Speed of the decision making. The graph shows the time in minutes (median and quartiles) until the minimum number of bees for a statistically significant decision was reached (p<0.05, MannWhitney U test). The data include trials with 24 and 64 bees.

We were also interested in whether or not there exists a correlation between group size and the speed of the decision making as a measure of scaling properties of the observed collective system. We evaluated groups of 24 and 64 bees and measured the time until the threshold for a statistically significant decision for the optimal temperature spot was reached. The time until the threshold is reached differed significantly: It took groups of 24 bees significantly longer (median = 12.16 minutes, n = 8 experimental runs) to reach the threshold for a clear decision for the optimum than groups of 64 bees (median = 4.53 minutes, n = 7 experimental runs)(Fig. 10; p<0.05, Mann-Whitney U test).

### Spatial Distribution of Honeybees in Homogeneous Thermal Environments

To examine whether the bees choose their location independently or in dependence of interaction with other bees we conducted experiments without any thermal gradient and compared the number of bees in the three different zones at the end of the experiments to the distribution predicted by the UDM.

**Figure 11:**
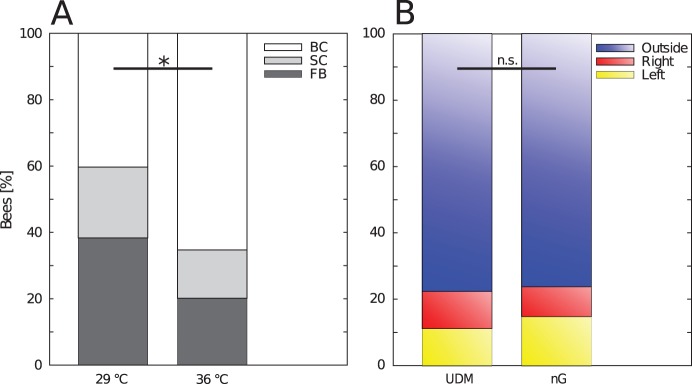
Cluster formation and distribution of bees at homogeneous temperatures. (A)shows the percentage of bees not in clusters (free bees, FB), bees in small clusters (2–3 bees, SC) and the bees in big clusters (>3 bees, BC) at 29±1°C compared to the free bees, bees in small clusters and bees in big clusters at 36±1°C (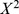-test p<0.05). The data include all groups sizes (24, 128). (B) shows the distribution of bees in the three different evaluation zones (left, outside and right) compared to the predicted distribution of the UDM (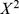-test p>0.05). The data include all groups sizes (24, 128) and temperatures (29±1°C, 36±1°C).

**Figure 12:**
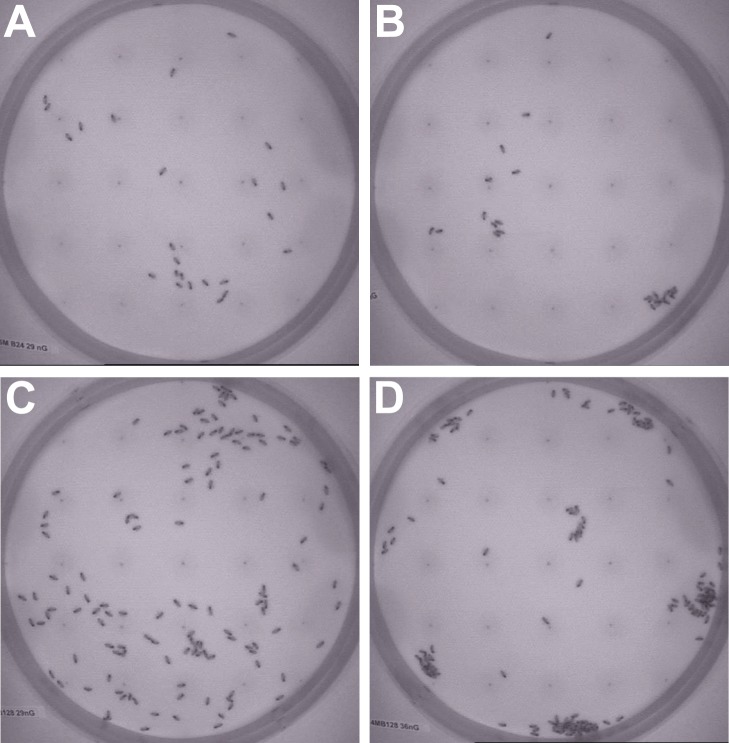
Exemplary pictures of experiments with homogeneous temperature distribution. Shown are pictures of the distribution of differently sized groups at different temperatures at minute 30: (A) 24 bees at 29±1°C, (B) 24 bees 36±1°C, (C) 128 bees 29±1°C, (D) 128 bees 36±1°C.

In 31 out of 37 trials, the bees did not distribute in an uniform distribution, thus we can reject the UDM for our data (p<0.05, 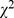 test). In trials with a homogeneous temperature of 29°C the percentage of single bees (38.2%) and small clusters (21.36%) was significantly higher than in trials with a homogeneous temperature of 36°C where the majority of bees was found in bigger clusters (65.3%) at the end of the experiment (see Fig. 11a, p<0.05, 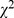 test). Without any temperature clue present in the arena (no-gradient condition), the location of these clusters are randomly chosen in a uniform distribution, as the pooled data of all bee locations across all trials was not statistically discriminable from the UDM (Fig. 11b, p>0.05, 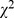 test). For pictures of the distribution of bees in exemplary experiments for the different temperatures and group sizes please see figure 12.

## Discussion

By investigating the collective aggregation behaviour in complex and heterogeneous thermal environments we gained novel and interesting insights into the social behaviour of honeybees, especially new thoughts concerning the organisation and behaviour of young honeybees in the brood nest. We can show that the collective aggregation behaviour is based on a self-organised process which is based on a modulation of the individual behaviour according to local environmental cues: after meeting another bee in the arena the bees wait longer the higher the local temperature is (Fig. 4). Our findings confirm the collective background of the observed thermotactic behaviour as most single bees do not locate themselves at the optimum while most of bees in trials with groups of bees aggregate there. The percentage of bees in the optimum is also significantly higher when the bees run in parallel within a group compared to the percentage of single bees (Fig. 6).

As we assumed, the bees neglected the sub-optimum in trials with groups of bees and showed a preference for the optimal spot (Fig. 7a). Our analysis showed that the thermotactic behaviour cannot arise from a simple gradient uphill walk of every individual bee: We introduced the bees in the centre of the arena, in the area with the lowest temperature, equidistant to the respective peak of the optimum and the sub-optimum. According to the AFM, purely uphill walking of every individual would lead to approximately a 60∶40 distribution of the bees between the optimal and the sub-optimal temperature spot (Fig. 6). In all tested group sizes a significantly higher percentage of bees was located at the optimal temperature spot than at the sub-optimal temperature spot (Fig. 7a,c) and the distribution of the bees deviated significantly from the prediction of the AFM (Fig. 6). Therefore we reject the hypothesis that the bees perform an uphill gradient walk to independently locate the optimum. Concerning the zonation of the bees the slightly increased percentage of bees in the sub-optimal temperature spot with increasing group size can be attributed to overcrowding in the optimal temperature spot (Fig. 7c). Some bees may switch to the sub-optimal temperature spot under crowded conditions: As bees can join aggregations only at the outer rim of the already aggregated group, the local temperature encountered by these late-arriving bees can be lower than the temperature encountered by the first arrivers in the centre of the sub-optimal temperature spot.

Inside a honeybee colony this relocation of young bees to an area with sub-optimal temperature within the brood nest could be adaptive (meaning beneficial for the survival probability of the colony by increasing the fitness) when we consider that the temperature in the brood nest is not homogeneous and the margins are cooler than the inner centre. In presence of a high number of other bees in the centre it would be advantageous for the colony that the young bees relocate and also perform their task of cell cleaning in the edge area, what would result in a homogeneous coverage of cell cleaning activity even under inhomogeneous temperature distributions within the brood nest and thus increasing the breeding success.

We also show that groups of young honeybees that are given the choice between two thermal optima of different attractiveness clearly decide for one of them collectively, provided that the group is big enough, thus the accuracy is affected by the group size. In our experiments only some of the small groups decided for one side collectively, so for the used arena size a minimum number of bees is needed to observe a significant collective decision at the end of a trial (Fig. 8a). The main reason for the lack of clear decisions in small groups could result from the diminished meeting probability of the bees, referring to the finding that the collective thermotaxis is based on the local interaction of bees: the resting time of the bees after meeting each other is positively correlated with the locally perceived temperature (Fig. 4). A similar behaviour leading to collective shelter selection was found in cockroaches: the resting time of a cockroach under a shelter is correlated to the number of other cockroaches there [39]. When groups make a decision, the vast majority decides collectively for the optimum (Fig. 8b). Thus we conclude from our experiments that the bees are able to collectively discriminate a local from a global optimum, even though the temperature in the optimal and sub-optimal spot fluctuates and even slightly overlaps during the decision making process (Fig. 2). This indicates that the collective decision making is a robust behaviour and is not strongly affected by little environmental changes. The robustness is also important for the colony, as the young cell cleaning honeybees should not disperse and leave the brood nest in the event of slight temperature fluctuations at their work site. In our experiment with the given arena size, groups of 6 bees were not large enough to successfully conduct this collective aggregation process. Thus, honeybees’ collective thermotaxis seems to be clearly density dependent. In turn, the density may also be an explanation for a minority of bigger groups deciding for the sub-optimum. Due to a high meeting probability and thus stopping frequency, the aggregation can get stable and attract more and more bees even in the sub-optimal temperature spot. This can also explain why it took smaller groups longer to reach the threshold for a statistically significant decision than bigger groups (Fig. 10): The meeting probability rises with group size, and thus bigger groups can form stable aggregations faster than smaller ones. So, both accuracy and the speed of decision making are density dependent. Comparable results were also found in collective decision making in fish shoals [40]. Surprisingly, when looking at the aggregation process there was no obvious difference between groups sizes (Fig. 9). The non-linear regression model (see equation 1) predicts that within a sufficiently long run time nearly all bees will be aggregated at the optimal temperature spot. The detailed analysis of the aggregation process over time, including the dispersal of the bees and the cluster formation, will thus be examined in future work.

Bees cluster together in natural environments, e.g., in winter clusters or reproductive swarms [41]–[43]. Studies concerning the cluster formation of bees showed that bees also cluster together in a darkened arena [44], [45]. For our experiments in homogeneous temperature gradients different outcomes were imaginable: The bees could for example either form many small cluster distributed all over the arena, or they could form only one or a few big clusters containing the majority of the bees at an arbitrary place in the arena. If one big cluster is formed without a temperature gradient present, the aggregation at the optimal temperature spot in expermiments with a complex gradient might happen by chance in some of the trials.

We can show that the clustering of the bees in the optimal temperature spot is not an occasional event. Our results from experiments with homogeneous temperatures indicate that the bees tend to form several small clusters scattered across the arena because although the location of th clusters deviates from the UDM when compared with every single trial the distribution of bees over all trials is not distinct from the UDM (Fig. 11). This way we can show that the individual honeybee is influenced by social cues due to the fact that the bees form clusters also in absence of a thermal gradient. As the overall distribution of clusters is not different from the UDM we further show that the position of the cluster in the optimum is not random. We conclude that the decisive cue for the collective thermotaxis is the locally perceived temperature. This can again be explained by the emergent aggregation behaviour. When two or more bees meet each other in the arena, the resting time correlates with the local temperature (Fig. 4), so the resting time should be equal in all areas with the same temperature, and thus the bees should join and leave the small aggregations constantly. This is also supported by our results form experiments with homogeneous temperatures, as the bees form more smaller clusters at 29°C, where the waiting time is short and form bigger clusters at 36°C where the waiting time is longer. We suggest that small temporary aggregations are formed all over the arena also in the first period of experimental runs with complex gradients. This way the aggregations formed by the bees in the optimum are an effect of the emergent collective thermotaxis in response to the individually perceived thermal environment. The bees should also not form one big cluster in an area of rather homogeneous temperature within the brood nest, but wander from one small aggregation to the other and thus rise the probability to reach all cells that require cleaning.

The analysis of the local interactions of the bees during the collective thermotaxis led to a simple algorithm for swarm robots, the BEECLUST algorithm [20]. It was tested under different environmental conditions in real robot swarms or simulations [20], [46]–[48] and some of our findings are comparable to the findings in artificial agents. For example, in a robot swarm which had to choose between dimmed and bright light as aggregation spots, the vast majority of the robots aggregated under the bright light, but some robots aggregated in the dimmed light when the space in bright light was crowded [46]. A sufficient number of robots is necessary for a collective decision, but a high density can also lead to jamming effects thus slowing down the aggregation process [48]. In the work presented here we did not conduct experiments with sufficiently big group sizes to observe such jamming effects, but it is known that jamming effects may occur on ant trails and function as a negative feedback in recruitment [49]. These examples indicate that the BEECLUST algorithm describes the underlying mechanisms of the bees aggregation behaviour in a sufficient way.

In consideration of all above discussed issues we can say that the collective thermotaxis we examined in this work is an example for a swarm intelligent behaviour. Millonas [50] listed some basic principles, from which several are applicable on our findings: *The proximity principle*: The group should be able to respond to an environmental stimulus. Our results show that the bees respond to the temperature gradient they are exposed to and show collective thermotaxis. *The quality principle*: The group should also be able to take into account the quality of an environmental factor. Our results demonstrate that bees are able to discriminate a local from a global optimum and to collectively choose the optimal temperature. *The principle of stability*: The groups should not switch the behaviour in response to every little fluctuation in the environment as this would consume more energy than it might bring in return. Our results show that the aggregation of bees in the optimum remains stable after the decision was made, in spite of little fluctuations of the temperature. In the work presented here we cannot give claim that the *principle of diverse response* and the *principle of adaptability* also apply to the examined swarm intelligent system, but those will be questions for future work.

In summary, groups of bees are not only able to form an aggregation collaboratively at a place with optimal temperature, but they are also able to distinguish between two different temperature optima and choose the better one collectively. We suppose that this behaviour is based on local interactions, without direct communication and global information, based on the positive correlation of waiting time and local temperature. Within the chosen constraints (e.g., groups sizes, shape of thermal gradient) of our experimental set-up, we could show that the behaviour is scalable and robust, as the group performance adjusts to different environmental conditions, but does not react to every little fluctuation and thus may be considered as very energy efficient, too. Although the abilities of each individual are limited, the collective thermotactic behaviour enables the bees to perform a complex task like the collective selection of a temperature optimum in a complex temperature gradient. Furthermore the bees exhibit typical swarm system characteristics, namely a correlation between group size and speed of the decision making or crowding effects. As mentioned, the behaviour of the bees is a strong inspiration for the field of swarm robotics. In turn, findings derived from robots can support biological hypotheses.

In future work we will further investigate the decision making process in honeybees (e.g., the flexibility of the collective choice, the cluster formation and scalability beyond the examined densities) in more complex and challenging experiments and also lay major focus on the roles of the individuals in the collective thermotaxis.
